# An integrated solar battery based on a charge storing 2D carbon nitride†

**DOI:** 10.1039/d2ee03409c

**Published:** 2023-02-15

**Authors:** A. Gouder, F. Podjaski, A. Jiménez-Solano, J. Kröger, Y. Wang, B. V. Lotsch

**Affiliations:** a Max Planck Institute for Solid State Research Heisenbergstr. 1 70569 Stuttgart Germany b.lotsch@fkf.mpg.de f.podjaski@imperial.ac.uk; b Department Chemistry, Ludwig-Maximilians-University Butenandstraße 5-13 81377 Munich Germany; c Departamento de Física, Universidad de Córdoba Campus de Rabanales, Edif. Einstein (C2) 14071 Córdoba Spain

## Abstract

Solar batteries capable of harvesting sunlight and storing solar energy present an attractive vista to transition our energy infrastructure into a sustainable future. Here we present an integrated, fully earth-abundant solar battery based on a bifunctional (light absorbing and charge storing) carbon nitride (K-PHI) photoanode, combined with organic hole transfer and storage materials. An internal ladder-type hole transfer cascade *via* a transport layer is used to selectively shuttle the photogenerated holes to the PEDOT:PSS cathode. This concept differs from previous designs such as light-assisted battery schemes or photocapacitors and allows charging with light during both electrical charge and discharge, thus substantially increasing the energy output of the cell. Compared to battery operation in the dark, light-assisted (dis)charging increases charge output by 243%, thereby increasing the electric coulombic efficiency from 68.3% in the dark to 231%, leading to energy improvements of 94.1% under illumination. This concept opens new vistas towards compact, highly integrated devices based on multifunctional, carbon-based electrodes and separators, and paves the way to a new generation of earth-abundant solar batteries.

## Abstract 2

We present an integrated solar battery with a bifunctional carbon nitride (K-PHI) photoanode, combined with polymeric hole transport and cathode materials. Light charging enables energy storage and increases battery round-trip efficiency by 94%.

## Graphical abstract

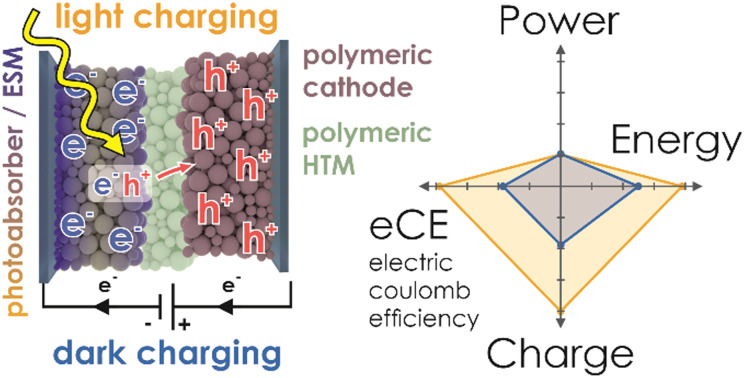

**Broader context**

Harvesting abundant solar radiation presents a very promising avenue to produce renewable energy, yet provides its own set of challenges: Stochastic fluctuations of the solar flux generate intermittency on timescales of months (winter-summer), days (day-night) or minutes to hours (e.g. weather), which requires energy storage functionalities to balance. The emerging concept of solar batteries incorporates light absorption functionality into batteries. Bifunctional photoanodes or –cathodes push this idea further by utilizing materials capable of both charge storage and light absorption, at the expense of a complex charge transfer mechanism. In this work, we present the earth-abundant carbon nitride K-PHI as bifunctional photoanode to simultaneously absorb light and store electrons, and design a new internal charge transfer mechanism *via* an organic polymer hole transporter as battery separator to enable both ion conduction and rectified photogenerated hole transfer to the cathode. Our internal mechanism resembling a planar heterojunction solar cell facilitates operation of the device and only requires low-cost earth abundant materials, both key for applications, which requires high levels of integration.

## 1. Introduction

While the world transitions from fossil to sustainable energy sources, integrating fluctuating renewable energy into the power grid provides its very own set of challenges. Volatile wind and solar energy suffer from intermittent availability; this requires enhanced flexibility of the power grid as well as new energy storage technologies. In particular, photovoltaics (PV) produces significant stochastic intraday fluctuations (*e.g.*, due to cloud overcast), which requires short-term energy storage solutions in the time range of minutes to hours.^1,2^ Energy storage technologies can help to balance this residual load.

Solar batteries and solar capacitors are a relatively new class of devices, which aim to integrate energy harvesting functionalities into energy storage devices.^3,4^ While discrete charging technologies are widely employed nowadays (*i.e.*, battery and PV are independent units that are connected as stacks or *via* DC–DC converters^5^),^6^ integrating PV and batteries into a single device is attracting increased interest due to its more facile implementation, flexibility, and volume minimization.^7^ Such devices can be categorized as three-electrode configurations (PV electrode, anode, and cathode) and two-electrode configurations (either bifunctional anode and cathode, or bifunctional cathode and anode). Three-electrode configurations were demonstrated using photoelectrodes inside of batteries^8–10^ or *via* different solar redox flow battery designs.^11–13^ Two-electrode configurations have been enabled *via* heterojunctions by depositing photoactive layers onto the anode of a battery or capacitor,^14^ or *via* composites of charge storage materials and photoactive materials.^15,16^

However, a third vista is gaining momentum: utilizing bifunctional photoelectrochemical energy storage materials, which are capable of performing both light absorption and charge storage in a single material.^17^ There are several reports of bifunctional materials, both inorganic (*e.g.* V_2_O_5_,^18,19^ MoO_3_,^20^ TiO_2_,^21^ or 2D perovskites^22^) and organic (*e.g.* covalent organic frameworks,^23^ quinone derivatives,^24^ or porous organic cages^25^). Notably, all existing device designs rely on transfer of the photogenerated charge carriers to the anode or cathode *via* an electrolyte or additional external circuit during charging, with the separator between the electrodes acting solely to conduct ions and prevent a short circuit, inspired by traditional battery designs, and thus resemble integrated PV-batteries. Note that three-electrode designs such as photo(super) capacitors also require an external charge transfer of photogenerated charge carriers and additional redox shuttles to close the internal circuit.^4,26,27^ Simultaneous photocharging and discharging *via* a load is complicated, since the external wiring is engaged in the charging process, and external electronics are necessary to change from charging to discharging mode. These drawbacks motivate us to investigate pathways of internal photogenerated hole transfer with a separator that simultaneously acts as hole shuttle.

We recently reported a bifunctional solar battery electrode material based on the fully earth-abundant 2D carbon nitride potassium poly(heptazine imide) (K-PHI).^17^ Upon light excitation (bandgap of ∼2.7 eV), electron–hole pair separation, and extraction of the hole, K-PHI can “trap” photoexcited electrons up to several hours^28,29^ to days^30,31^ and release them on demand, accompanied by a color change from yellow to blue. This combination of optoelectronic and optoionic properties, which are linked to photointercalation of K^+^ ions^32^ and electron trapping in an intercalation band within the bandgap produces (pseudo)capacitive electron storage^17,29^ and has led to applications in “dark” photocatalysis,^33–37^ photomemristive sensing,^29^ and multifunctional light-driven microswimmers.^38,39^

**Fig. 1:**
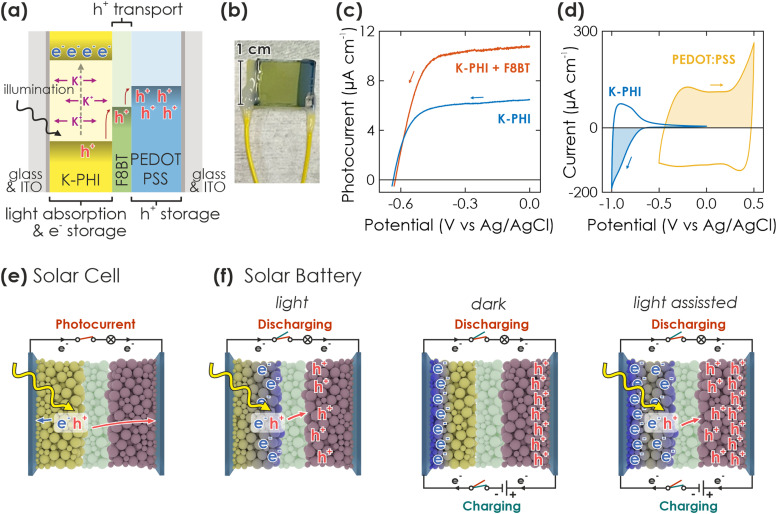
Concept and requirements of a solar battery device. (a) Scheme of a direct solar battery device, comprising K-PHI as photoactive and electron storage material, the HTM F8BT and the HSM PEDOT:PSS, sandwiched between two ITO sheets. The hole transport process is indicated with red arrows. (b) Picture of a direct solar battery device. The left and right wires are soldered to the substrate which is in contact with K-PHI and PEDOT:PSS, respectively. (c) Linear scanning voltammetry (LSV) curves of K-PHI (blue) and K-PHI decorated with the HTM F8BT (red), measured in an aqueous electrolyte containing the sacrificial electron donor methanol (100 mM) in 3-electrode configuration. K-PHI + F8BT shows a significantly larger photocurrent at potentials more positive than −0.4 V *vs.* Ag/AgCl, evidencing enhanced photogenerated hole extraction rates with the HTM. (d) Cyclic voltammetry (CV) measurements of ITO electrodes containing K-PHI (blue) and PEDOT:PSS (yellow), measured in 3-electrode configuration. (e and f) Schemes of different operation modes of the device, either as solar cell (e) or solar battery with various operation modes (f). Yellow and blue balls represent K-PHI in its respective discharged and photoreduced charged state, green balls refer to the HTM, and red balls represent the HSM. The blue rectangular cuboids represent the ITO substrate. Black lines show external wire connection and current flux direction during different operations. We show the charging mechanism of the solar battery *via* only light (left), only electric (middle) or simultaneously using light and electric (right) power simultaneously. Color code of the switches defines the circuit switches in respective operations (red when extracting charges from the device (either as photocurrent or *via* discharging), green when charging the battery). Note that both circuits utilize the same connection on the device, but are plotted on top and bottom of the device to visualize different operations.

Herein, we design a proof-of-concept “direct solar battery” using K-PHI as active layer, which is tasked with absorbing light and storing photoexcited electrons as well as balancing charges with intrinsic K^+^ ion movement. Contrary to currently published designs discussed above, we do not use an external circuit to transfer holes to the hole storage material (HSM) counter (electrode), but rather rely on an internal ladder-type hole transfer cascade performed by a multifunctional hole transport material (HTM) – a design more reminiscent of planar heterojunction-type solar cells than of batteries (Fig. 1(a)). Photocharging occurs internally under open circuit potential (OCP) conditions. We first identify suitable materials, poly(9,9-dioctylfluorene-*alt*-benzothiadiazole) (F8BT) as HTM and poly(3,4-ethylendioxythiophene) polystyrene sulfonate (PEDOT:PSS) as HSM, and then investigate kinetics and performance of different operation modes: (i) charging *via* illumination only and under open circuit potential (OCP) conditions, (ii) solely electric in the dark with an external current, and finally (iii) in a light-assisted electric mode.

## 2. Results and discussion

### 2.1. Device design

The concept of the solar battery is visualized in Fig. 1(a): K-PHI absorbs light and generates electron–hole pairs. Charge separation likely occurs close to the junction to the hole acceptor,^34,40^ which can either be a redox shuttle or hole transport material (HTM). A solid HTM presents fewer self-discharge pathways *via* an electrolyte (*e.g.*, water oxidation or reduction) and is less prone to recombination since charges are immediately shuttled to the hole storage material (HSM) – a problem which has been identified as major challenge for solar batteries.^3^ In the integrated direct solar battery we propose herein, we use a solid HTM, which acts as a battery separator and redox shuttle to transport photogenerated holes from K-PHI to the HSM, while mobile ions (K^+^, H^+^) provide internal electrostatic charge compensation (Fig. 1(a), green for HTM (F8BT) and blue for HSM (PEDOT:PSS)).^17,41–43^ Simultaneously, the HTM acts as a rectifier and prevents self-discharge *via* an internal short-circuit between K-PHI and HSM (*i.e.*, if holes are not only shuttled from K-PHI to the HSM, but also back from the HSM to K-PHI to quench the electrons trapped on K-PHI). The solar battery can be discharged on demand *via* an external electric circuit. Note that this approach with a multifunctional HTM separator is thus far unique since it does not require an external wire to shuttle charge carriers from one electrode to another during charging, further facilitating implementation by allowing simultaneous light charging and electric discharging, as well as operation as a solar cell.

We now discuss the fabrication of the device and the rationale behind the materials selection (Fig. 1(b)): Films of K-PHI on indium tin oxide substrates (ITO) were prepared according to a procedure recently described by us.^17,29^ In brief, K-PHI was synthesized in a salt melt containing KSCN and the 1D heptazine based polymer melon.^35,44,45^ Subsequently, the product was washed, exfoliated *via* sonication in isopropanol, and homogeneous films of 0.5–2 μm thickness were obtained *via* dip coating (see Methods section and ESI,† Section S1 for more details). In order to optimize charge separation at the K-PHI/HTM interface, we first performed screening experiments of both conductive polymer and small molecule HTMs (deposited *via* spin coating onto K-PHI) utilizing the sacrificial electron donor methanol as a replacement for the HSM (see ESI,† Section S2 for a more detailed discussion).^17^ By evaluating photocurrent as a figure of merit, we identified F8BT as the most suitable candidate. The linear sweep voltammogram of K-PHI and F8BT, measured against a reference electrode in three-electrode configuration, is shown in Fig. 1(c) under 1 Sun illumination and compared to bare K-PHI. At a potential of 0 V *vs.* Ag/AgCl where photogenerated electrons are discharged, an oxidative photocurrent of 10.7 μA and 6.47 μA is reached for K-PHI with and without F8BT, respectively, highlighting the beneficial role of F8BT to extract holes from K-PHI. With more negative potentials, the photocurrent decreases nearly linearly due to a decreasing driving force for electron extraction, until at −0.4 V *vs.* Ag/AgCl it collapses to 0 μA. For all samples, we observed an open circuit potential (OCP) of about −0.6 V *vs.* Ag/AgCl (Fig. 1(c)). We explain the increase in photocurrent upon addition of the HTM by an improvement of the photogenerated hole extraction efficiency – a step which is known to be limiting for carbon nitride photo(electro)catalysts^29^ – which in return decreases recombination of photogenerated charge carriers and increases the photocurrent response, leading to better photocharging of K-PHI. As HSM, we chose the widely studied conductive polymer PEDOT:PSS, deposited analogous to F8BT *via* spin coating. PEDOT:PSS was shown to operate as a p-type substrate capable of reductively quenching holes on n-type K-PHI upon photoexcitation. This process is akin to photocharging and underlines the suitability of PEDOT:PSS as HSM.^40^ Charge storage is enabled at potentials more positive than the valence band of K-PHI and F8BT *via* a well investigated pseudocapacitive mechanism, making it a suitable cathode material.^46^ The ladder-type redox band position (band alignment) of the HTM and HSM (Fig. 1(a)) is thus suitable for extracting photogenerated holes in a cascade process. Three-electrode cyclic voltammetry (CV) measurements of K-PHI and PEDOT:PSS samples in the dark show the charge storage potential and capacity of both materials (see overlay in Fig. 1(d)). While K-PHI shows its well-reported typical CV shape with a charging onset at −0.65 V *vs.* Ag/AgCl,^17^ PEDOT:PSS produces a nearly rectangular CV – typical for its pseudocapacitive charge storage mechanism.^47^ Note that the capacity of PEDOT:PSS is chosen larger than of K-PHI to prevent a performance bottleneck on the cathode side. Utilizing all these components allows us to realize the integrated solar battery (Fig. 1(b)).

### 2.2. Operation modes of the device

A solar battery can be operated in different modes.^3,7^ Upon illumination, the resulting photocurrent can be accessed by connecting the anode and cathode to the potentiostat and applying a suitable bias voltage. The device then operates analogous to a solar cell (Fig. 1(e)). However, when operating under OCP conditions (*i.e.*, no current is extracted *via* the current collectors), the photogenerated electrons and holes accumulate in the anode and cathode and thus, charge the device (Fig. 1(f), left). Subsequently, the stored charges can be accessed in the dark by applying a suitable discharge current until the cell voltage reaches 0 V (voltage at which the device is fully discharged).

Conversely, we can also perform electric charging and discharging in the dark *via* an external current (*i.e.*, a current applied *via* the potentiostat) – analogous to a galvanostatic charging and discharging experiment (GCD) in a normal battery (Fig. 1(f), middle). The capacity depends on the charging and discharging current rates and voltage window. The latter should be estimated from the photovoltage measured during light charging. Notably, when illuminating the device during a GCD experiment, the current flux is created by both light generated charges and the electric charging *via* the potentiostat, thus maximizing performance (Fig. 1(f), right). The overall effective charging current is the sum of both applied “external” current and “internal” photocurrent from light absorption. We will discuss these different operation modes in the following.

#### 2.2.1. Solar cell operation

**Fig. 2:**
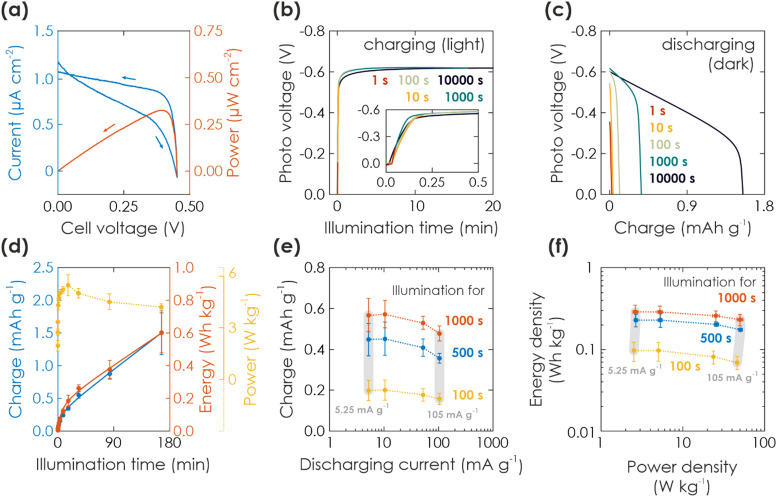
Solar battery characterization of light charging process. (a) Current–voltage (blue) and power (red) curves (10 mV s^−1^) of a solar battery sample in solar cell mode, illuminated with 1 Sun. (b) Charging of the solar battery with different illumination times at 1 Sun and under OCP conditions (inset shows a zoom of short illumination times). (c) Subsequent electric discharging in the dark with a fixed current of 10.5 mA g^−1^ (normalized against mass of K-PHI, HTM, and HSM). (d) Gravimetric capacity, energy, and power density extracted from the charging *via* illumination (b). (e) Kinetic study of the discharging process. Charging is performed *via* illumination for 100 s (yellow), 500 s (blue), and 1000 s (red). Subsequent immediate discharging is carried out with different discharging currents (5.25, 10.5, 52.5, 105 mA g^−1^; smallest and largest current shown with vertical grey bar). (f) Ragone plot displaying the energy and power output with increasing illumination times and same discharging currents given in (d). Vertical grey bars links dots measured at the same smallest and largest discharge current.

We first evaluate performance of the solar battery when operated as a solar cell. A device is immersed into oxygen-free 0.1 M KCl electrolyte and an initial activation measurement is performed. Activation measurements are necessary to remove all unwanted charges from both anode and cathode, which might reside on the sample from synthesis (see ESI,† Section S3 for details) and affect device characterization. Subsequently, we illuminated the sample with an LED (365 nm, 100 mW cm^−2^) and performed a CV measurement between OCP and 0 V cell voltage with a slow scan rate of 10 mV s^−1^ to simulate quasi-static conditions. The voltage sweeps are shown in Fig. 2(a). At 0 V, we measured a short circuit current (*I*_SC_) of 1.07 μA cm^−2^ g^−1^ on the backwards voltage sweep (from OCP to 0 V). Note that the mass of the device is calculated from the measured mass of K-PHI, HTM, and HSM. With increasing potential, the photocurrent decreases and at a potential of approximately 0.40 V it collapses to 0 V. The OCP is 0.45 V and maximum power of 0.326 μW cm^−2^ is reached at a current of 0.828 μA cm^−2^ and a voltage of 0.39 V, resulting in a fill factor (FF) of 0.73 (all values calculated from backwards sweep). This behavior is in principle also observed when illuminating with a 365 nm LED (100 mW cm^−1^) increasing the photon flux that can be absorbed, albeit at higher absolute currents (FF of 0.70, see ESI,† Section S9). While this FF is considered to be high for organic solar cell devices and is larger than common solar cells incorporating carbon nitrides as dyes,^48,49^ the losses result from the small but significant slope of the current between 0 V and 0.30 V. This slope is probably caused by high series and low shunt resistance (*e.g.*, due to pinholes, or traps^50^) as well as a decreasing driving force for charge separation. In case of a solar battery, current increase or loss due to a partial charging of K-PHI and PEDOT:PSS seems also possible (*vide infra*). The hysteresis between positive and negative voltage sweeps further indicates such a behavior: the larger current on the backward voltage sweep (*i.e.*, from OCP to 0 V) might be a convolution of photocurrent and discharging current of K-PHI. Such a behavior is desirable for the solar battery light charging modes discussed next.

#### 2.2.2. Solar battery operation *via* light charging

We can also employ the internal photocurrent to charge the device in lieu of extracting it immediately as discussed in the previous section. This enables the most characteristic function of a solar battery: its ability to charge solely *via* illumination. We demonstrate in an experiment charging under illumination and electric discharging in the dark in Fig. 2(b) and (c): After immersing a solar battery sample into a degassed 0.1 M aqueous KCl electrolyte and performing the activation measurement (see ESI,† Section S3), we illuminated the device from the backside for a given time at 1 Sun under OCP conditions. Note that the task of the 0.1 M KCl electrolyte in the reactor is only to provide an oxygen-free environment, ensure stable temperature during illumination, provide sufficient humidity and to facilitate reset measurements (see ESI,† Section S3). A photovoltage of 0.6 V developed during the first 50 s and remained constant during the ensuing illumination. Subsequently, the light was turned off and the device was discharged at a current of 10.5 mA g^−1^ until the cell voltage dropped back to 0 V. Electric discharging in the dark for increasing illumination charging times (1s to 10 000 s) is shown in Fig. 2(c). The shape looks similar to a typical GCD battery measurement and indicates a faradaic charge storage mechanism: A plateau-like potential decrease to *ca.* 0.5 V for short and 0.3 V for long illumination times, followed by a sharp voltage drop to 0 V. Note that the plateau has a certain slope <0, indicating pseudocapacitive contributions of the charge storage mechanism, and possibly also caused by increasing K-PHI or cell resistance upon discharging.^17,29,47^ We discuss the charge storage mechanism more thoroughly *via* a kinetic analysis with CV measurements in ESI,† Section S4.

The respective capacity, energy, and power output is plotted against the illumination time in Fig. 2(d). Energy output can be calculated with 
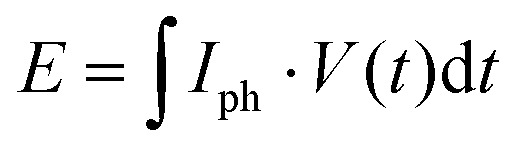
, with *I* being discharge current and *V*(*t*) being the cell voltage during discharging. Average power is calculated with *P = E*·*t*^−1^, with *t* being the discharging time. When illuminating the sample for 10 000 s, we can extract a charge of 1.5 mA h g^−1^ and an energy of 0.60 W h kg^−1^. A saturation behavior of the capacity becomes evident for illumination times above *ca.* 2000 s (Fig. 2(d), blue: initial slope is decreasing), but is not reached after 10 000 s, suggesting that the charging process is likely slowed down by the concomitant charge accumulation^29,51,52^ limiting photocharging efficiency and that the true capacity of K-PHI is hence larger. It is noteworthy that the shape of the extracted charge and energy curves are similar; this is because energy output depends on the photovoltage *V*(*t*), which is almost constant at illumination times where the plateau is reached (>100 s; see Fig. 2(d), yellow). Utilizing the energy data, a maximum solar-to-output efficiency of 0.012% can be calculated when taking only light energy larger than the bandgap of K-PHI into account (see ESI,† Fig. S5.2). If energy of the whole solar spectrum is considered, the solar-to-output efficiency is reduced to 0.002%. Power output for these illumination times is approximately constant since it is governed by the photovoltage as well. We discuss self-discharge in ESI,† Section S8 and show a charge retention of 72% after 1000 s, when the device is illuminated for 1000 s.

Next, in order to analyze discharging kinetics, a solar battery sample was illuminated with 1 Sun for three representative durations (100, 500, 1000 s) under OCP conditions and subsequently discharged in the dark with different current densities (5.25, 10.5, 52.5, 105 mA g^−1^). The extracted charge is shown in Fig. 2(e). A smaller discharging current results in a larger capacity as common for batteries due to less diffusion limitations and less resistive losses resulting from the intrinsically low conductivity of K-PHI.^17,43^ While we observe this larger capacity with lower currents for all illumination times, it is more pronounced for longer durations (when comparing currents of 5.25 and 105 mA g^−1^: 26% and 19% larger capacity for the smaller discharge current at illumination times of 100 s and 1000 s, respectively). Scaling of energy and power density with current is presented *via* a Ragone plot in Fig. 2(f): energy density behaves analogous to the capacity (ESI,† Fig. S6.1), *i.e.*, a minor increase is observed for smaller currents (5.25–10.5 mA g^−1^). With illumination duration the energy density increases for all currents (Fig. 2(f): yellow to red) – analogous to the behavior of energy and charge scaling with illumination time discussed above. Power density increases with current, since the cell voltage for different discharge currents is approximately constant (see GCD profiles in ESI,† Fig. S5.1 and respective power output in ESI,† Fig. S6.1), in line with the discussion of power output dependence on illumination time above. Thus, the kinetic behavior results in a Ragone plot with rather horizontal lines, which scale upwards (*i.e.*, increase in energy density) with illumination time, highlighting the device's energy-stable operation at larger discharge currents, *i.e.*, increased power density (up to approx. 10 W cm^−2^ kg^−1^). We will discuss comparison to energy storage and solar battery devices in more detail in Section 2.2.3 in the context of light-assisted electric charging and discharging.

#### 2.2.3. Solar battery operation *via* electric and light-assisted electric charging

So far, we have discussed the ability of the solar battery to charge *via* illumination. However, as a second pathway to modify the charging state, analogous to a normal battery we can apply an external electric charging current, which can be combined with the internal photocurrent to store both solar and electric energy simultaneously in one device. To differentiate between different illumination modes during GCD, we use the terminology “cEdE” to describe electric charging and discharging in the dark, “cLEdE” when illuminating during electric charging, and “cLEdLE” when illuminating during both charging and discharging. Cell efficiency metrics defined by parameters such as capacity, energy and power density, or electric coulombic efficiency (eCE) can significantly improve by these combined methods. The eCE gives the ratio between charges being electrically discharged and electrically charged and thus, presents a metric to evaluate charge gains *via* illumination (for cEdE: eCE is analogous to the coulombic efficiency). In the following, we will discuss solar battery operation modes which include electric charging *via* an applied current to modify the charging state.

**Fig. 3:**
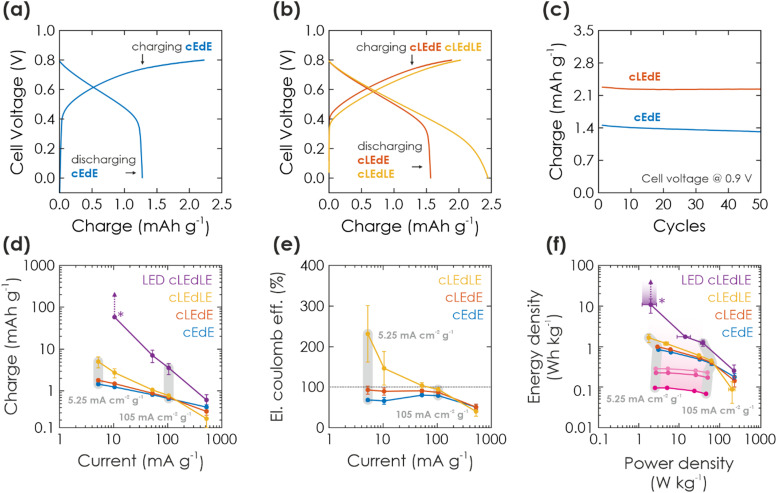
Solar battery characterization of electric and light-assisted electric charging process. (a and b) A cycle showing GCD in the dark (a) (cEdE) and under 1 Sun illumination (b) during charging (cLEdE) or during charging and discharging (cLEdLE) with a current of 10.5 mA g^−1^. (c) Extracted charge in cEdE and cLEdE mode and a cell voltage window of 0.9 V, plotted against the cycle number. (d) Extracted charge as a function of electric charging and discharging current, when operating the solar battery in a voltage window of 0.8 V and cEdE, cLEdE or cLEdLE modes. Note that LED-cLEdLE is analogous to cLEdLE, but with a 365 nm LED (power analogous to 1 Sun: 100 mW cm^−2^) providing the illumination. The “*” marks a data point, where discharging could not be completed and the measurement was aborted (due to the photocurrent being larger than the discharging current). The vertical arrows show how charge would have increased with a later aborted measurement. The two exemplary vertical gray bars show measurements at the same charging and discharging current. (e) Electric coulombic efficiency for the measurement shown in (d), highlighting the performance gains from illumination. (f) Ragone plot, showing energy and power output at the currents given in (d) and (e), underlining how illumination can push the device performance to larger energy and power values. Charging solely *via* illumination is extracted from Fig. 2(e) and shown in purple, with illumination times increasing from 100 s to 1000 s from dark to bright purple. Arrow and “*” mark again the aborted measurement (as discussed in (d)).

We first perform GCD with a current of 10.5 mA g^−1^ from 0 to 0.8 V in similar conditions as for the light charging measurements discussed above (see ESI,† Section S7 for the rationale behind the chosen voltage window), but in the dark (cEdE mode). We show a cycle in Fig. 3(a). For charging, we observe an initial fast voltage increase to about 0.5 V, followed by an area with significantly slower voltage increase (*i.e.*, “plateau” region). When discharging, we observe a similar trend: slow voltage decrease from 0.8 V to 0.5 V and a subsequent collapse to 0 V. Charging and discharging requires on average 580.2 s and 417.9 s, respectively. Note that the shape of the discharge curve looks similar to when the sample is only charged *via* illumination for short illumination times (Fig. 2(c); albeit at *ca.* 0.2 V smaller voltage), hinting onto a similar discharging mechanism.

Light-assisted GCD can be performed in cLEdE (Fig. 3(b), red) and cLEdLE (Fig. 3(b), yellow) operation modes. Compared to the dark measurement (Fig. 3(a)), for charging we observe again an initial region of fast voltage increase to about 0.5 V and a subsequent “plateau” region with a slope comparable to the dark measurement. However, for discharging the “plateau” lasts longer to about 0.4 V for cLEdE and 0.2 V for cLEdLE, analogous to charging with long illumination times (Fig. 2(c)). Charging and discharge requires on average 523.3 s and 520.0 s for cLEdE as well as 608.8 s and 1395 s for cLEdLE, respectively. This results in more extracted charge, which we show in Fig. 3(c) for a cell voltage of 0.9 V: Capacities of both dark and light GCD measurements are compared for 50 cycles, yielding an initial capacity of 1.4 mA h g^−1^ and 2.3 mA h g^−1^ (92% and 98% retained after 50 cycles), respectively. To explain mechanistic differences in electric charging *via* GCD (discussed here) and purely light charging (discussed in Section 2.2.2), we assume that GCD charges K-PHI and PEDOT:PSS close to the current collector (ITO substrate) first. In contrast, photocharging requires interfacial charge separation and therefore occurs rather at the junction to the HTM, or at least more in the bulk of the active layer of the battery (visualized in Fig. 1(f): compare position of electrons in left *vs.* middle panel). Since photogenerated electrons close to the HTM junction have to travel through the bulk of the K-PHI layer to discharge *via* the substrate, charge transport limitations due to the internal resistance of K-PHI (*i.e.*, larger iR drop) will have a larger effect as compared to electrons injected close to the substrate. Thus, self-discharge for light charging must be larger than for electric charging, which we show and discuss in ESI,† Section S8. Besides affecting discharging kinetics, the larger iR drop for photocharged electrons reduces the final cell voltage (compare cell voltage at the end of the discharging plateau in cEdE (0.5 V, Fig. 3(a)), cLEdE and cLEdLE (0.4 V and 0.2 V, Fig. 3(b))). Thus, simultaneous charging *via* light and the substrate allows to access more of the solar battery volume on short time scales, which benefits the overall capacity and thus energy density. In addition, illumination increases the material's conductivity,^17^ thereby facilitating also the electric charging process.

To deepen our understanding of the influence of light on parallel electric charging, we perform a kinetic study by changing charging and discharging currents. In Fig. 3(d), we show the respective charge output for cEdE, cLEdE, and cLEdLE modes (LED-cLEdLE is analogous to cLEdLE, but uses a 365 nm LED (100 mW cm^−2^) as light source). Note that measurements were performed in a smaller voltage window of 0.8 V compared to 0.9 V used for the cycling stability discussed above (Fig. 3(c)) due to the more stable operation in this voltage window at small currents and under illumination, allowing for a more reliable comparison of different operation modes. When operating in cEdE mode, with smaller currents (5.25–10.5 mA g^−1^) the capacity increases as the system is kinetically less limited. At the same time, this increase starts to saturate for very small currents due to the longer discharging time invoking self-discharge (a discussion on self-discharge after electrical charging is given in ESI,† Section S8). When operating in cLEdE mode (Fig. 3(d), red), the change of capacity behaves analogous to the dark case, but with an offset to larger capacities (compared to the dark case: at a current of 5.25 mA g^−1^ we observe an increase of extracted charge of 22.0% to 1.79 mA h g^−1^). The offset results from the internal photocurrent assisting charging the device as discussed above. This effect is more pronounced for smaller currents (at a current of 105 mA g^−1^ we could only observe an increase of 5.54%), which we explain with the longer charging time resulting from small currents leading to an elongated illumination time. When operating in cLEdLE mode (Fig. 3(d), yellow), the overall illumination time becomes much longer since the internal photocurrent is also continuously generated during discharge, which in return significantly increases the extracted charge output (compared to the dark case: at a current of 5.25 mA cm^−2^ g^−1^ we have observed an increase of extracted charge of 243% to 5.02 mA h g^−1^). Note that when the internal photocurrent is in the range of or larger than the external discharging current, the extracted charge increases very significantly (see cLEdLE in Fig. 3(d) for small currents) or even rises into infinity. We demonstrate the latter by providing illumination *via* a LED at similar illumination power compared to solar simulators with 1 Sun (100 mW cm^−2^), but which only illuminates at wavelengths of *ca.* 360–375 nm where K-PHI can absorb (Fig. 3(d), LED-cLEdLE). IV curves of illumination *via* 1 Sun (Fig. 2(a)) and 365 nm LED are compared in ESI,† Section S9. At a discharge current as low as 10.5 mA g^−1^, we could never complete discharging due to strong continuous internal photocurrent generation and had to abort the measurement after extracting a charge of 20 mC (58.4 mA h g^−1^). Hence, the observed increase in extracted charge (243%, see Fig. 3(d)) is larger than for reported devices with a similar bifunctional electrode, but different device designs (57% and 95% increase in capacity for V_2_O_5_ photocathodes for lithium- and zinc-ion batteries^18,19^). This increase in charge should not be confused with an increase of capacity of the battery, but rather demonstrate a beneficial operation mode of a solar battery due to continuous charge generation under illumination. The capacity of K-PHI was reported to be 25.6 mA h g^−1^, the equivalent of 1 electron per every 4th heptazine unit.^31^ Nevertheless, the theoretical capacity of K-PHI is likely larger, especially when charged electrically to higher potentials than possible by bandgap excitation.

Next, we compare the eCE for the different solar battery operation modes (Fig. 3(e)), a metric which we define as the ratio between electric charging and electric discharging. Note that in comparison to the coulombic efficiency (CE), eCE can exceed 100% since charge generation stemming from the internal photocurrent is not taken into account in the electric external charging current.^23,24^ In cEdE mode, we reach the maximum eCE (here the same as CE) of 80.7% at a current of 52.5 mA g^−1^. Larger as well as smaller currents produce a decreased eCE due to kinetic limitations and self-discharge, respectively.^17^ The eCE value is in fact larger than for K-PHI in a half-cell configuration reported by us earlier (approx. 72%^17^), which we explain with less self-discharge *via* the inevitable aqueous electrolyte of half-cell measurements (enabling water reduction *via* uncovered parts of the substrate). When operating in cLEdE, we can alleviate the eCE for small currents since additional charging occurs *via* the photocurrent. Thus, this mode allows a more efficient operation of the solar battery in a region where the capacity is larger (*i.e.*, smaller currents), with a maximum eCE of 92.9% (for cEdE: 68.3%) at a current of 5.25 mA g^−1^. When operating in cLEdLE, we see a significant increase in eCE for small currents, with a maximum of 231% at a current of 5.25 mA g^−1^. Analogous to our rationale behind the increase of extracted charge discussed above, we explain this behavior with a significantly longer illumination time compared to cLEdE, resulting in much more photocharging. Thus, the eCE reported here for cLEdLE is much larger than for literature reports of 112%.^24^ Note that eCE is not a cell efficiency, since it only takes into account electron flux (*i.e.*, current) into and out of the cell and does not include the incoming photon flux, which is responsible for the photocurrents and hence, additional charges measured.

Finally, we compare power and energy density for different currents *via* a Ragone plot (Fig. 3(f)). The cEdE measurement resembles the behavior of a normal battery:^47^ Maximum energy density (0.846 W h kg^−1^) for small power at small currents and maximum power density (62.3 W kg^−1^) for small energies at large currents. This behavior is caused by kinetic limitations of the discharging process, as discussed above. When operating in cLEdE or cLEdLE mode, we observe a similar curve shape as the dark case, but with an offset to larger energy densities (compared to cEdE at maximum energy: 16.0% and 94.1% increase to 0.982 W h kg^−1^ and 1.64 W h kg^−1^, respectively). This effect is more pronounced for smaller currents and can be explained analogous to our abovementioned rationale for larger capacities and improved eCE with internal photocurrent assisting in charging of the bulk. Power density slightly decreases concurrently due to the altered GCD discharging profile (compare Fig. 3(a) and (b)). Increasing illumination intensity *via* an LED (*ca.* 360–375 nm, 100 mW cm^−1^) significantly amplifies the energy enhancement: Simultaneous to our discussion on extracted charge, we had to abort the measurement after extracting 10.8 W h kg^−1^ since the discharging process could not be completed, but upon longer operation the energy output should approach infinity, as indicated by the purple arrow in Fig. 3(f).

To understand the origin of performance improvements in the Ragone plot for cLEdE, cLEdLE, and LED-cLEdLE mode, we show the performance of charging solely *via* illumination as discussed in Section 2.2.2 in the same plot (Fig. 3(f), purple): Longer illumination times lead to more photocharging, *i.e.*, vertical scaling in the Ragone plot. Illumination assisted GCD measurements discussed here are affected in an analogous manner, *i.e.*, energy increases with illumination time. Power density on the other hand scales with current and is more or less independent of illumination duration, when not taking the effect of altered GCD shapes (Fig. 3(b)) into account. Thus, its scaling in the Ragone plot looks similar for all operation modes including charging solely *via* illumination (compare Fig. 3(f) data points at different currents, marked with gray bars). A direct comparison of energy and power scaling with discharge current for all operation modes shows the similar scaling best and is given in ESI,† Fig. S6.1.

## 3. Conclusion

**Fig. 4:**
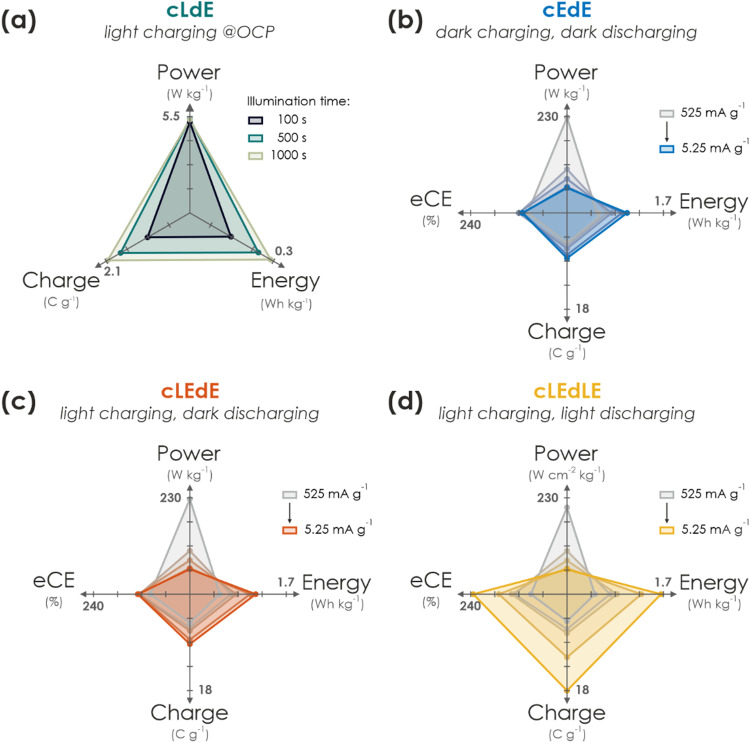
Performance summary of operation modes of the solar battery. (a) Pure photocharging at OCP and discharging in the dark (cLdE) at a constant current of 10.5 mA g^−1^. While power output remains approximately constant, charge and energy scales with illumination time. (b) GCD in the dark (cEdE) with different current densities (current decreases from grey to blue). With decreasing currents, power decreases and energy, charge as well as eCE slightly increases. (c) Same plot as in (b) for electric charging with different currents, but under illumination (1 Sun) during charging for additional photocharging (cLEdE). Discharging is performed with the same current in the dark. Power, energy, charge, and eCE scale with current akin to (b), but with slightly enhanced performance. (d) Same plot as in (b) and (c), but under illumination during both charging and discharging (cLEdLE). While power scales analogous to (b) and (c) with current, energy, charge and eCE is significantly enhanced thanks to the solar cell output during both charging and discharging, increasing the device performance significantly compared to when operated in the dark.

In this work, we have presented a proof-of-concept integrated solar battery device based on the earth-abundant carbon nitride K-PHI, which serves as both light absorber and charge storage (photo)anode. Photogenerated holes in K-PHI are shuttled *via* a HTM (F8BT) to the HSM (PEDOT-PSS) *via* an interfacial hole transfer cascade. This device can work as a solar cell; however, its capabilities exceed those of a solar cell: When kept under OCP (*i.e.*, no current is applied) the generated internal photocurrent charges the photoactive material and the HSM. No external wiring is required for this charging process. Subsequently, the charge can be accessed by applying a suitable discharge current. We also demonstrate purely electric charging *via* a charging current as a second path of accumulating charge on the device and also combine both modes, resulting in light-assisted electric (dis)charging that boosts the device performance further. *via* kinetic studies, the performance limitations and metrics of the device are discussed while showing how Ragone plots can be used and behave for such devices. We summarize performance parameters for different modes and kinetics in Fig. 4. A comparison to literature solar battery devices is given in Table S10.1 (ESI†), which suggests that our solar battery device favorably compares with other solar batteries utilizing bifunctional photoanodes.

An important message of this work is to provide a fundamental understanding of the solar battery operations as a convolution of different ingoing or outgoing energy fluxes, which impact the charging state. The charging contributions are as follows: (i) energy input *via* illumination depends on the generated internal photocurrent, which itself relies on the material's absorption profile, incident photon flux and illumination time (Fig. 4(a)). (ii) Energy input *via* electric charging in the dark (Fig. 4(b)) emulates a classic battery (cEdE), *i.e.*, the capacity increases with lower charging and discharging currents due to smaller kinetic limitations. We show that a combination of light and electric charging during either charging (cLEdE) or both charging and discharging (cLEdLE) yields a performance enhancement in terms of apparent discharge energy, apparent capacity, and eCE. Thus, we provide an energy storage device, the apparent performance output of which can be tuned *via* illumination (see Ragone plot in Fig. 3(f)) and relies on a double functionality of light absorption and electron storage in a single material. The device design based on a ladder-type internal hole transfer cascade, which so far is the first of its kind for solar batteries, renders it a closer relative to solar cells than to classic batteries. It thus establishes a new generation of direct solar batteries derived from solar cells with bifunctional (both light absorbing and charge storing) components. Designing multifunctional polymeric separators in opens new avenues to solar battery devices that utilize both photogenerated electrons to improve the overall charging efficiency and photogenerated holes to efficiently shuttle them to the HSM. Our approach presents a cost and material efficient alternative route to circumvent oxidative reactions with the electrolyte, which has been identified as a key challenge for solar batteries.^3^ In addition, the presented design based entirely on earth-abundant electrode materials makes production and application facile (*i.e.*, no external circuit is required for the charging process, simultaneous light charging and electric discharging is possible) and underscores the application potential of this new concept of truly integrated solar batteries based on bifunctional materials, especially where low cost and high levels of integration are key (for example in autonomous microsystems, self-powered sensors, or solar battery parks).

## 4. Experimental

### 4.1. Synthesis of K-PHI & anode preparation

K-PHI was synthesized as described in literature *via* a salt-melt of potassium thiocyanate (KSCN) and melon.^33,44,45^ Precursors KSCN and melamine were acquired from Sigma Aldrich in reagent grade purity. Exfoliation was performed *via* sonication in an ice bath for 2 h in a 2-propanol (IPA) suspension (Sigma Aldrich) with a concentration of 3 mg mL^−1^. Nanosheets were separated *via* two consecutive centrifugation steps (353 RCF for 20 min, then 795 RCF for 40 min; 3–30k, Sigma), akin to reported procedures.^17,29^ Density was evaluated by drying 1 mL of suspension and subsequently weighing the residue. Particle concentration was increased to 0.3 mg mL^−1^ by evaporating the respective amount of IPA *via* a rotary evaporator. Films were deposited onto the transparent conductive substrate indium tin oxide (ITO; Ossila Ltd.) *via* dip coating (*ca.* 400 dips, 100 mm min^−1^ extraction speed, 2 min drying between dips; Rotary dip coater, Nadetech), yielding films of approx. 500 to 1000 nm.

Subsequently, for depositing the HTM a solution of F8BT (Ossila Ltd.) in chloroform with a concentration of 10 mg mL^−1^ was spin coated (2000 rpm for 30 s; WS-650MZ-23NPP, Laurell) onto the K-PHI film and annealed for 10 min on a hot plate at 80 °C. This process was repeated to increase the HTM film thickness. The sample was contacted by scratching off a small part of both films at a corner of the sample to uncover the substrate and by gluing a wire to it with conductive silver paste (Silver Conductive RS 186-3600, RS-Pro). The contact was then sealed with epoxy glue (DP410, 3M Scotch-Weld) to provide both a rigid connection and prevent the silver paste as well as ITO to participate in the measurements.

### 4.2. Cathode preparation

For the cathode, an aqueous suspension of PEDOT:PSS with a concentration of 3–4 wt% (Sigma Aldrich) was spin coated (2000 rpm, 30 s; WS-650MZ-23NPP, Laurell) onto an ITO substrate (Ossila Ltd) with dimensions of 10 × 12 mm, which underwent plasma cleaning with oxygen plasma (Femto, Diener) for 10 min prior to deposition to both clean the surface and make it more hydrophilic. Subsequently, the sample was annealed for 20 min at 145 °C in a nitrogen atmosphere. This process was repeated to increase PEDOT:PSS film thickness to approx. 600 nm. The samples was then contacted analogous to the anode as described in the previous section.

### 4.3. Fabrication of the solar battery full cell

First, adsorbed oxygen was removed from both anode and cathode half-cell samples by applying vacuum and subsequent argon for 6 cycles using Schlenk techniques. Both samples were then sandwiched onto each other (with a contact area of 1 cm^2^), a weight of approx. 15 g was put on top to provide enough pressure, and epoxy (DP410, 3M Scotch-Weld) was applied on the two opposite edges to generate a sturdy connection, while leaving the two other faces open to enable contact with the surrounding electrolyte. Subsequently, after drying of the epoxy the sample was immersed into a degassed aqueous 0.1 M KCl solution. The mass of the device was calculated by weighting the K-PHI/HTM sample after film deposition on the substrate and calculating the mass of the PEDOT:PSS sample from measured film thickness and density of dried films reported by the manufacturer.

### 4.4. (Photo)electrochemical measurements

All electrochemical measurements were performed in a photo(electrochemical) reactor equipped with a quartz glass for illumination of the respective sample, and with a multichannel potentiostat (Autolab M204, Metrohm). An aqueous solution containing 0.1 M potassium chloride (KCl; Sigma Aldrich) was used as background electrolyte (for experiments requiring a sacrificial electron donor, the respective donor was added as well), which was degassed with argon (>99%) prior to every measurement to ensure oxygen free conditions. For three-electrode-measurements, we have utilized an Ag/AgCl reference electrode with a saturated KCl electrolyte (RE-1CP, ALS Japan) and a gold foil (Sigma Aldrich) counter electrode. For two-electrode-measurements, the sample was directly connected to the potentiostat. Illumination was provided either with a calibrated solar simulator (LightLine A4, Sciencetech), providing artificial sunlight according to AM 1.5G with class AAA quality, or with a LED at 365 nm equipped with a collimator (M365LP1-C4, ThorLabs) and set to a power output of 100 mW cm^−2^.

## Author contributions

AG, FP, AJS, JK, and BVL conceived the project. AG performed the measurements. AG and FP, with assistance of YW, analyzed the data. FP, AJS, and BVL supervised the research. AG and FP wrote the manuscript with assistance of all authors.

## Conflicts of interest

The authors declare no conflict of interest.

## Supplementary Material

EE-016-D2EE03409C-s001
