# Supplementary material for: An integrated solar battery based on a charge storing 2D carbon nitride
Source: Energy Environ Sci. 2023 Feb 15;16(4):1520–30. doi: 10.1039/d2ee03409c (PMC10091497; doi:10.1039/d2ee03409c)
Supplement: EE-016-D2EE03409C-s001 [file EE-016-D2EE03409C-s001.pdf]

Electronic Supplementary Material (ESI) for Energy & Environmental Science.
This journal is © The Royal Society of Chemistry 2023

Supplementary Information

An Integrated Solar Battery based on a Charge Storing 2D Carbon Nitride

AUTHOR LIST AND AFFILITATION

A.Gouder[1,2], F. Podjaski[1,3]*, A. Jiménez-Solano[1,4], J. Kröger[1], Y. Wang[1], B. V. Lotsch[1,2]*

1: Max Planck Institute for Solid State Research, Heisenbergstr. 1, 70569 Stuttgart, Germany

2: Department Chemistry, Ludwig-Maximilians-University, Butenandstraße 5-13, 81377 Munich, Germany

3: Current address: Department of Chemistry, Imperial College of London, London, W12 0BZ, United Kingdom

4: Departamento de Física, Universidad de Córdoba, Campus de Rabanales, Edif. C2, 14071 Córdoba, Spain

CONTACT

*Corresponding authors: b.lotsch@fkf.mpg.de, f.podjaski@fkf.mpg.de

Table of content

1. Morphological characterization 2
2. Electrochemical study of HTM materials 3
3. Activation & reset of solar battery samples 4
4. Kinetic analysis of charge storage mechanism 6
5. Additional analysis: Light charging and electric discharging 7
6. Additional analysis: Scaling of Power and Energy with Discharge Current 9
7. Shape of GCD measurements & chosen voltage window 10
8. Charge retention 12
9. Solar cell performance comparison 1 sun & 365 nm LED 13
10. Comparison of battery performance with literature 14
11. References 15

1. Morphological characterization

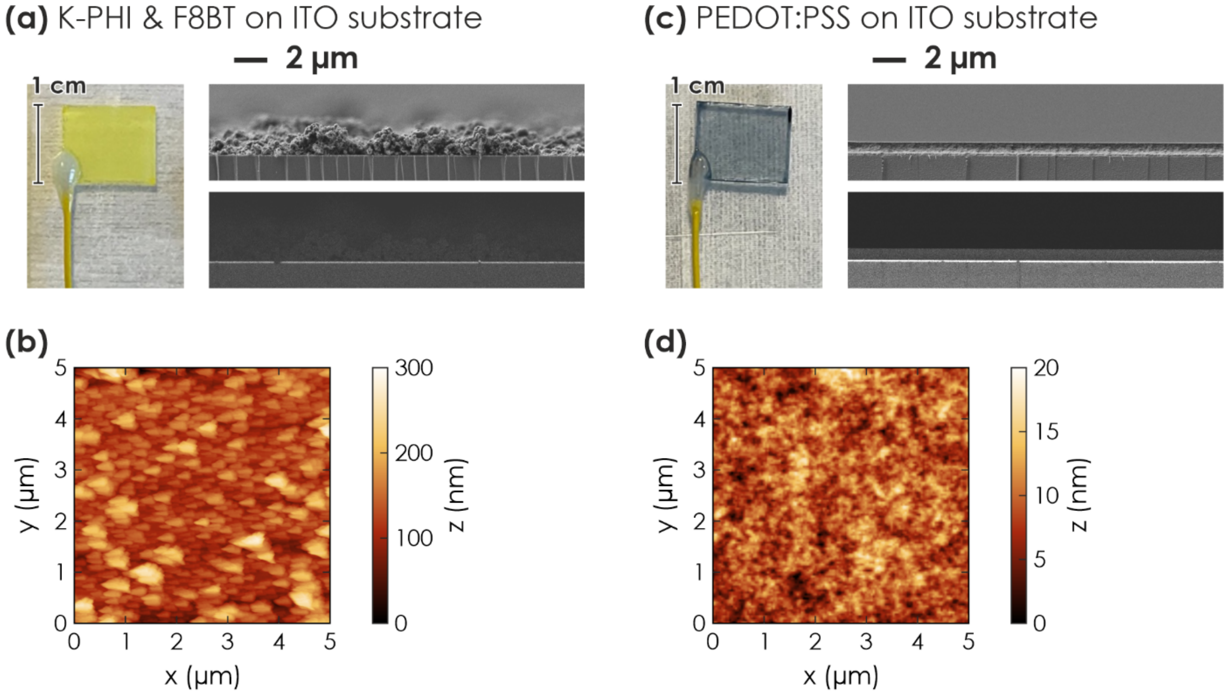

Fig. S1.1 | Morphological characterization of films via SEM and AFM. (a) Characterization of the K-PHI film functionalized with F8BT (picture of a sample on the left) via SEM of a cross section. Secondary electron detector (right, top) and backscattered electron detector (ESB) (right, bottom) images are recorded, showing a rough surface with an average film thickness of ca. 1 µm. **(b)** AFM image of the film shown in (a). A root mean square roughness (RMS) of 35.8 nm is calculated. **(c)** Characterization of the PEDOT:PSS film via SEM of a cross section. Secondary electron detector (right, top) and backscattered electron detector (ESB) (right, bottom) images are recorded, showing a smooth surface with an average film thickness of ca. 0.6 µm. **(d)** AFM image of the film shown in (c). A root mean square roughness (RMS) of 2.59 nm is calculated.

2. Electrochemical study of HTM materials

The list of suitable hole transport materials (HTM) is vast, both for small molecule HTMs and conductive polymer HTMs. We chose multiple candidates based on their suitably positioned valence band potential and other desirable characteristics, such as conductivity, size or other functionalities (e.g., a band gap, which could allow them HTM to participate as active material). Conductive polymers (poly(3-hexylthiophen-2.5-diyl) (P3HT), Poly[4,8-bis(5-(2-ethylhexyl)thiophen-2-yl)benzo[1,2-b;4,5-b']dithiophene-2,6-diyl-alt-(4-(2-ethylhexyl)-3-fluorothieno[3,4-b]thiophene-)-2-carboxylate-2-6-diyl)] (PTB7TH), Poly(N-vinylcarbazole) (PVK), Poly(9,9-dioctylfluorene-alt-benzothiadiazole) (F8BT)) and small molecule (N,N'-Bis(3-methylphenyl)-N,N'-diphenylbenzidine (TPD), 1,3,5-Tris(N-carbazolyl)benzene (TCP), Tris-(4-carbazoyl-9-yl-phenyl)-amin (TCTA)) HTMs were chosen and deposited onto K-PHI films (on a transparent conductive substrate (here ITO) via dip coating) via spin coating. A solution of HTM in chloroform (1 mg mL^{-1}) was prepared, spin coated at 2,000 rpm for 30 s and subsequently annealed for 10 min at 80 °C on a hot plate.

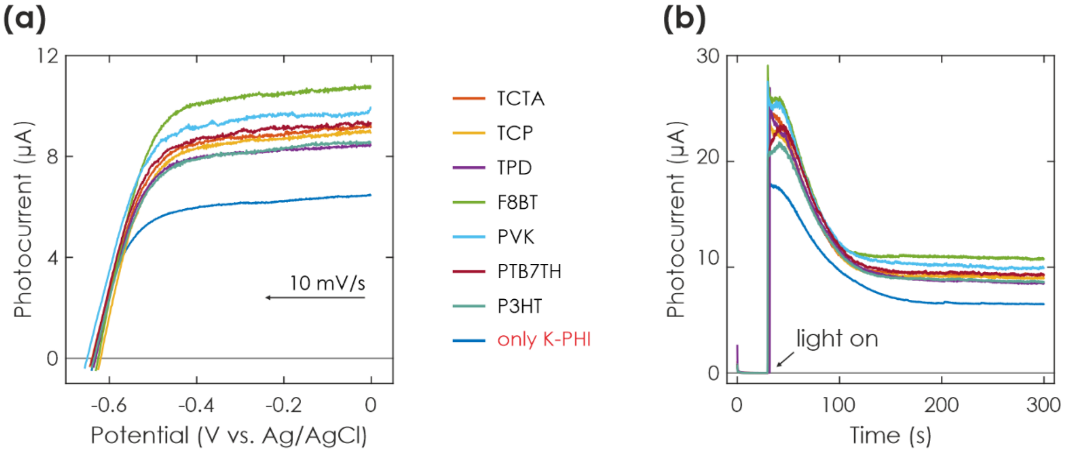

Fig. S2.1 | Electrochemical characterization of K-PHI samples with different HTMs. (a) LSV measurements of K-PHI with respective HTMs in a 3-electrode configuration and under illumination. All HTM materials show a larger photocurrent than the K-PHI sample alone, with F8BT showing the largest improvement. **(b)** Photocurrent output, when applying a bias voltage of 0 V to the device. An initial large photocurrent is observed, which stabilizes after ca. 100 s onto a "steady state" photocurrent.

To evaluate the performance of HTM materials, the photocurrent at different potentials was evaluated in a 3-electrode CV measurement, in the presence of a sacrificial electron donor to replace the HSM and under illumination with 1 sun. In Fig. S2.1 (a), the IV curves of the sample with different HTMs are shown. The potential is swept into the negative direction at a scan rate of 10 mV s^{-1}. From 0 to about -0.4 V vs. Ag/AgCl, the photocurrent is very slowly decreasing due to a reducing driving force of the electron extraction from K-PHI. Subsequently, an open circuit potential (OCP) of approx. -0.65 V vs. Ag/AgCl can be measured for all samples. F8BT shows the largest photocurrents and has thus been chosen for the further development of the device.

3. Activation & reset of solar battery samples

Prior to each solar battery measurement, an activation measurement was performed to ensure comparability between different samples. An exemplary measurement routine is shown in Fig. S3.1.

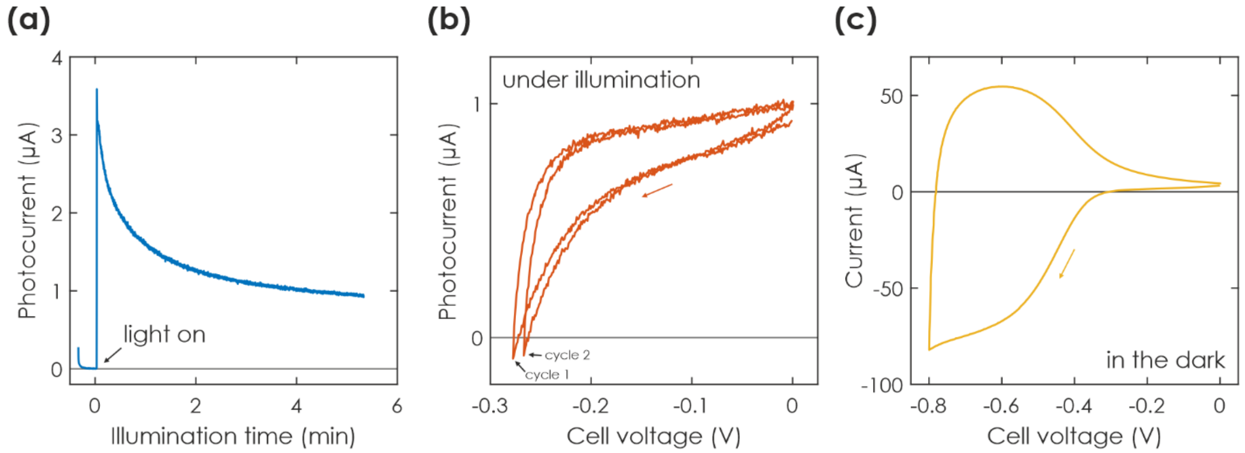

Fig. S3.1 | Activation measurement routine. (a) Photocurrent when applying a bias voltage of 0 V to the solar battery device. The bias was applied for 5 min. **(b)** CV under illumination and between 0 V and OCP, cycled twice with a scan rate of 10 mV s^{-1}. **(c)** CV in the dark, cycled once with a scan rate of 50 mV s^{-1}.

First, a bias voltage of 0 V was applied under illumination for 5 min. This ensures that possible organic contaminations are oxidized by K-PHI. Subsequently, a CV was measured under illumination between 0 V and the OCP (i.e., as soon as a cathodic current is observed). The idea behind this measurement is to control whether the sample shows the typical IV curve and is not short circuited (e.g., through accidental pinhole formation during sample preparation). Finally another CV measurement was carried out in the dark between 0 V and -0.8 V to check that both K-PHI and PEDOT:PSS work as intended when charged electrically in the dark.

Subsequently, a reset measurement was performed to remove all charges left on the device, i.e., electrons from K-PHI and holes from PEDOT:PSS, and to ensure that the sample was in a state that is comparable to other samples at different stages of the measurement. This measurement was performed both after the activation discussed in the paragraph above and after GCD measurements. An exemplary measurement is shown in Fig. S3.2.

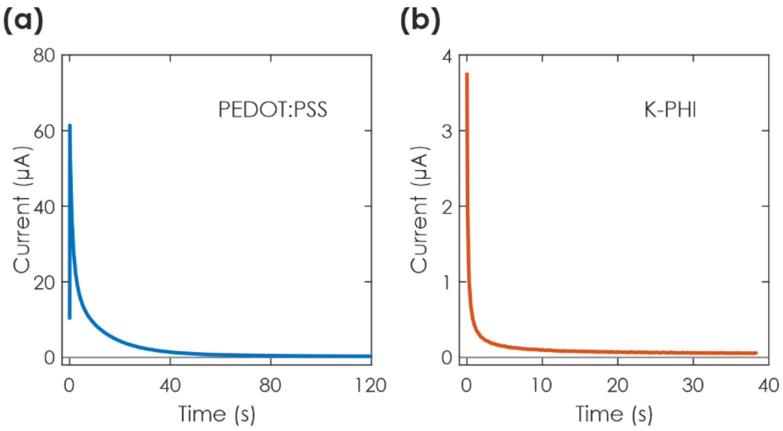

Fig. 3.2 | Reset measurement routine. An exemplary reset measurement is shown for PEDOT:PSS **(a)** and subsequently K-PHI **(b)**. A bias potential was applied to the respective electrodes of the device and a bias potential of -0.1 V vs. Ag/AgCl (PEDOT:PSS) and 0 V vs. Ag/AgCl (K-PHI) was applied. The measurement was stopped as soon as the current reached a value < 50 nA.

The concept behind this measurement is to remove unwanted charges from the electrodes individually, i.e., first remove holes from PEDOT:PSS, then electrons from K-PHI. It ensures that the charge state (or "oxidation state") of K-PHI and PEDOT:PSS is in balance prior to each measurement. For this, only one electrode was connected as working electrode, and a bias potential of 0 V vs. Ag/AgCl was applied against a reference electrode and a counter electrode which were immersed in the same electrolyte as the sample. The potential was applied until the current reached a value of < 50 nA. This measurement was first done for PEDOT:PSS and then for K-PHI.

4. Kinetic analysis of charge storage mechanism

In this section, we present a more detailed kinetic analysis of the charge storage mechanism of the two half cells (i.e., K-PHI and PEDOT:PSS; measured in a 3-electrode setup in a degassed 0.1 M KCl electrolyte against an Ag/AgCl reference electrode and Au counter electrode) as well as the full cell via a scan rate dependent CV analysis. Results are shown in Fig. S4.1.

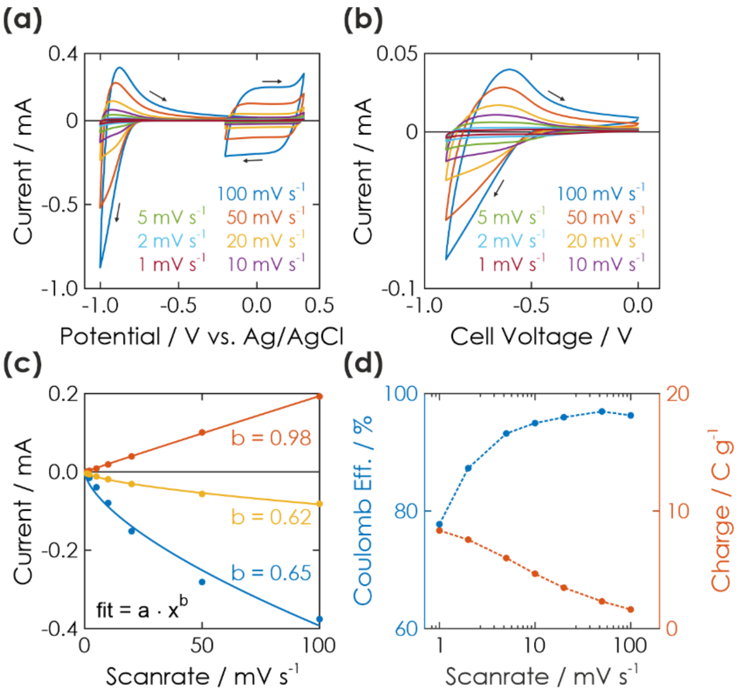

Fig. S4.1 | Kinetic study via CV and with different scan rates. (a) Study of K-PHI between 0.0 and -1.0 V vs. Ag/AgCl, and PEDOT:PSS between 0.5 and -0.1 V vs. Ag/AgCl for different scan rates. **(b)** Study of a full cell solar battery sample between 0 and -0.9 V vs. Ag/AgCl for different scan rates. **(c)** Extracted current of half cells ((a): K-PHI in blue, extracted at -0.9 V vs. Ag/AgCl; PEDOT:PSS in red, extracted at 0 V vs. Ag/AgCl) and full cell ((b), at -0.9 V, yellow) in dependence of the scan rate. A fit was calculated using the formula in the inset.[1] **(d)** Extracted charge and coulombic efficiencies from CV full cell measurements shown in (b). Normalized onto mass of K-PHI, HTM, and PEDOT:PSS

We will first discuss the half cells. K-PHI shows its very specific CV shape (Fig. S4.1 (a), the curve at more negative potentials). When extracting the current at the anodic sweep peak and plotting it against the scan rate, the data can be fitted according to the equation in the inset of Fig. S4.1 (c). The exponent b of 0.65 unveils largely faradaic or pseudocapacitive charge storage kinetics,[1, 2] in line with our previously reported results.[3] On the other hand, when extracting current of the PEDOT:PSS measurement at a potential of 0 V vs. Ag/AgCl, the fit reveals a much more linear dependence of current with scan rate (b value of 0.98). Since this is more reminiscent of a capacitive kinetic signature,[4] but the charge storage mechanism of PEDOT:PSS is known to be faradaic, we link the kinetic behavior to pseudocapacitve charge storage,[1, 2, 5, 6] as widely reported for PEDOT:PSS.[7-10]

Next, when looking at the scan rate dependence of peak current of the full cell (Fig. S4.1 (b)) and fitting the data (Fig. S4.1 (c)), a b value of 0.62 can be calculated, indicating a faradaic or pseudocapacitive charge storage mechanism. Note that this value is similar to the K-PHI half-cell, suggesting that the kinetics of charge storage of the full cell is dominated by K-PHI photoanode, which thus also limits the performance of the device. When looking at the extracted charge (Fig. S4.1 (d)) as a function of scan rate, we observe that with slower scan rates more charge can be extracted. This indicates that the amount of charge is limited by the charging kinetics, i.e., a very slow charging and discharging process maximizes the effective capacity – analogous to GCD measurements discussed in the main text in Fig. 3 (d). When looking at the coulombic efficiency as a function of scan rate of the full cell (Fig. S4.1 (d)), we see a maximum of 90 % at a scan rate of 50 mV s^{-1}. For both larger and smaller scan rates the coulombic efficiency decreases. We explain this with the existence of an optimum scan rate determined by kinetic limitations for larger scan rates and increased self-discharge for smaller scan rates, as previously reported for the K-PHI half cell.[3]

5. Additional analysis: Light charging and electric discharging

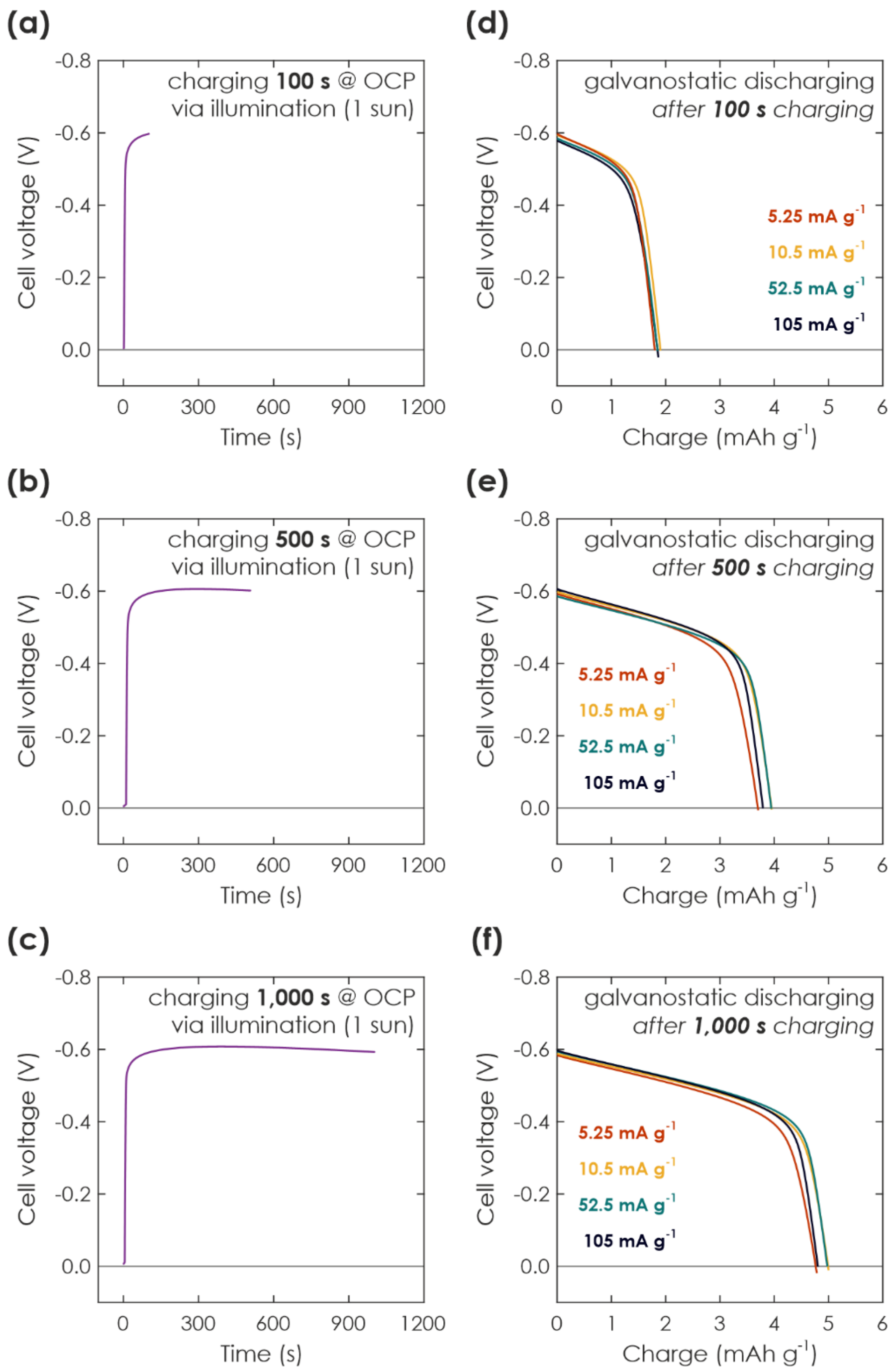

Fig. S5.1 | Kinetic study of light charging and subsequent immediate electric discharging, discussed in the main text in Section 2.2.2. (a-c) Charging of a solar battery sample via 1 sun illumination for three different illumination times. The solar battery sample is kept under OCP conditions to prevent any electric current during the light charging process. A photopotential of -0.6 V develops for all illumination times. **(d-f)** Respective electric discharging in the dark and with different currents given in the legend.

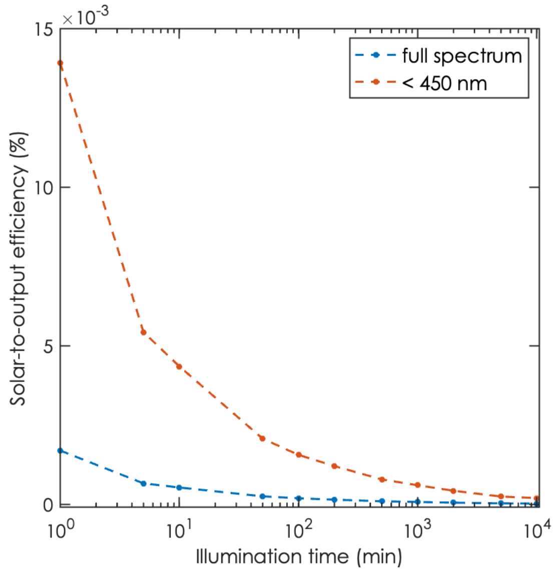

Fig. S5.2 | Calculated solar-to-output efficiency. The solar-to-output efficiency was calculated from light charging and electric discharging measurements shown in Fig. S5.1 as a function of illumination time. The light energy was calculated according to AM1.5 G 1 sun illumination, with either the full spectrum or only wavelengths below the bandgap (2.7 eV, corresponding to 450 nm).

6. Additional analysis: Scaling of Power and Energy with Discharge Current

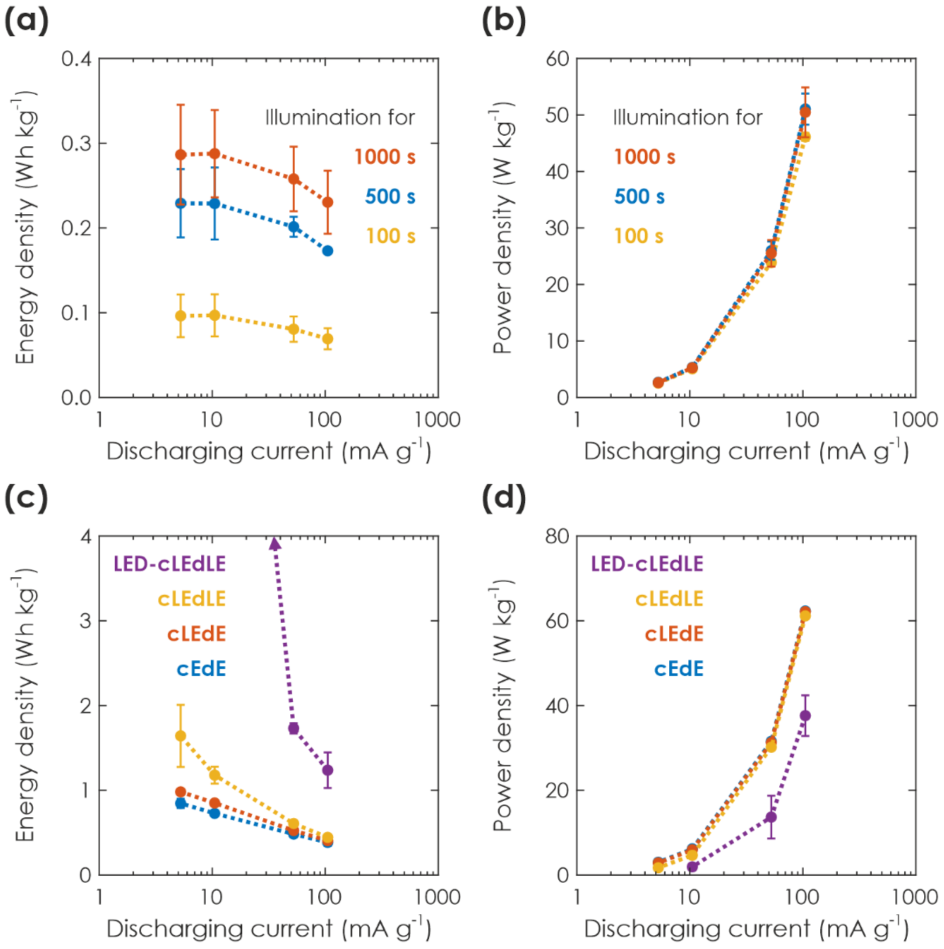

Fig. S6.1 | Additional analysis to main text Fig. 2 & 3. (a-b) Charging of a solar battery sample via 1 sun illumination and subsequent electric discharging in the dark at different discharging currents. Scaling of energy (a) and average power (b) with discharging current. The corresponding Ragone plot is shown in the main text in Fig. 2 (f). **(c-d)** Charging of a solar battery sample via GCD in the dark (cEdE), charging under illumination (cLEdE) or both charging and discharging under illumination (cLEdLE when illuminated with the solar simulator and 1 sun (100 mW cm^{-2}), or LED-cLEdLE when illuminated with an LED at 365 nm (100 mW cm^{-2})). Scaling of energy (a) and Power (b) with discharging current. The corresponding Ragone plot is shown in the main text in Fig. 3 (f).

7. Shape of GCD measurements & chosen voltage window

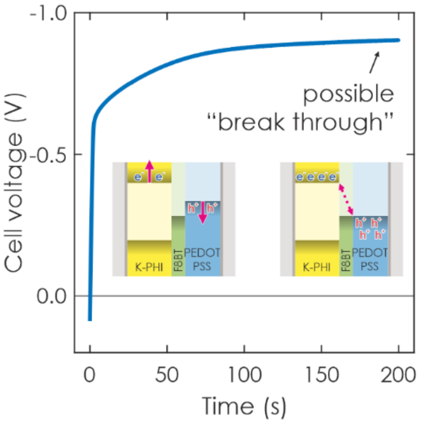

Fig. S7.1 | Analysis of shape of GCD curve during charging. GCD measured under analogous conditions as electric and light assisted electric GCD performance analysis discussed in the main text in Section 2.2.3 and shown in Fig. 3. The only difference is that the abort condition of charging is not the upper voltage vertex of 0.8 V. Instead, the measurement is stopped after 200 s. Inset shows normal charging (left) and a proposed short circuit or "breakthrough" mechanism (right).

In the following we will discuss the rationale behind the chosen GCD voltage window. The shape of the charging curve for a typical battery shows an initial fast increase to a relatively flat plateau in which the battery experiences charging. Subsequently after reaching a fully charged state, the potential increases rapidly again.[1, 4] Conversely, in our solar battery device we do not observe this second rapid increase. Instead, the plateau seems to flatten out (Fig. S7.1). We propose a possible mechanism for this behavior in the inset in Fig. S7.1: Electrons from K-PHI can reach holes from PEDOT:PSS, a process that is normally inhibited by the HTM F8BT and termed by us as "breakthrough" voltage. This is possible as soon as the potential of hole storage in PEDOT:PSS (i.e., the valence band of PEDOT:PSS) gets more negative than the valence band of F8BT. Consequently, holes are being injected from PEDOT:PSS into F8BT and can then recombine with electrons from K-PHI.

Note also that while photocharging of the solar battery occurs at a potential of 0.6 V (see main text Fig. 2 (b) and (c)), we reach a higher voltage of 0.8 V before the "break through" voltage is reached. We explain this by the different charging mechanisms of light charging via an "internal" photocurrent and electric charging via applying the "external" charging current. While the first charges the K-PHI film most likely more in the bulk and close to the junction to the HTM, the latter charges the film via the substrate. The low conductivity of K-PHI produces an iR drop across the film, which in return reduces the voltage at which the device is charged and subsequently discharged. This means: K-PHI charging starts at 0.6 V, and charges can be theoretically stored on the device until it reaches the "break through" voltage. However, while electric charging with a very small current should in theory stay at a similar potential than light charging and yield a similar capacity (note the bend in the cell voltage at ca. 0.6 V, when charged electrically as shown in Fig. S7.1), this current would produce a much smaller power output. Hence, we chose 0.8 V as a compromise for electric charging, which – while charging the device unevenly (i.e., the parts of K-PHI close to the substrate are charged more than the bulk) – produces a reasonably high power and energy output at currents between 5.25 and 105 mA g^{-1}. Note that in theory the capacity of K-PHI is thus underestimated in this work.

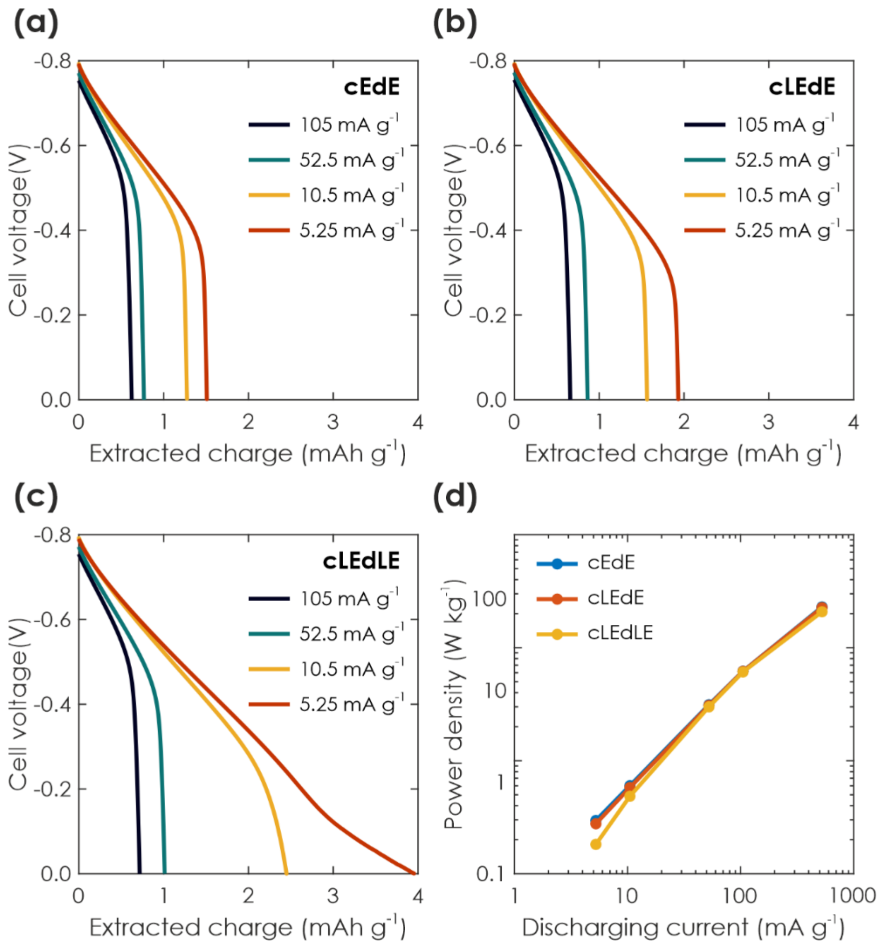

Fig. S7.2 | Analysis of shape of GCD curve during discharging. (a-c) GCD discharge profiles with different discharge currents and in the dark (cEdE) (a), illuminated during only charging (cLEdE) (b) or illuminated during both charging and discharging (cLEdLE) (c), measured in analogous conditions than measurements shown in the main text in Fig. 3 (d), (e), (f). **(d)** Power density of solar battery samples when GCD is performed in cEdE (blue; extracted from (a); hidden behind red curve), cLEdE (red; extracted from (b)), or cLEdLE (yellow; extracted from (c)). Power output reproduced from the main text Fig. 3 (f).

Next, we will address the shape of GCD discharge curves under different illumination conditions as shown in Fig. S7.2 (a-c). Conditions are analogous to measurements shown for GCD in the main text in Fig. 3 (d)-(f). Note that for cLEdLE and slower currents after discharging the plateau, the cell voltage does not rapidly decrease to zero but rather shows a tail. This is due to the photocurrent generated during illumination which simultaneously charges the device during electric discharging. It affects small electric discharging currents more since they are in the range of the photocurrent, as discussed in the main text. The power density output is thus affected and decreases for small discharging currents when discharged under illumination (Fig. S7.2 (d)).

8. Charge retention for delayed discharge

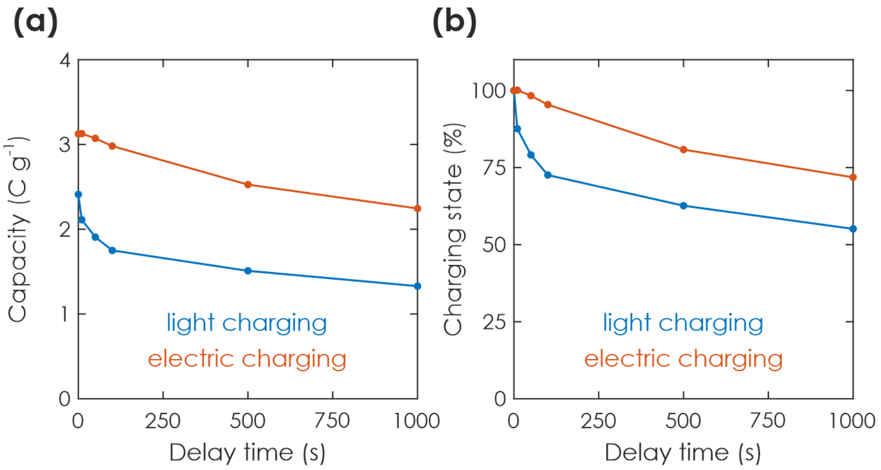

Fig. S8.1 | Analysis of charge retention. (a) Extracted charge from a solar battery, which was charged via illumination at OCP and discharged in the dark with 10.5 mA g^{-1} (blue), or charged and discharged in the dark (between a cell voltage of 0 V (discharged) and 0.8 V (charged)) with 10.5 mA g^{-1} (red). The subsequent electric discharge in the dark (10.5 mA g^{-1}) was delayed as shown in the Fig.. (b) Extracted charge retention from (a), normalized against the extracted charge without any time delay before the discharge.

An important feature of solar battery device is its charge retention time, i.e., how fast the solar battery self-discharges. To analyze this, we perform a measurement analogous to light charging and electric discharging measurements discussed in the main text in Section 2.2.2, but fix the illumination time onto 1000 s and instead delay dark discharging (10.5 mA g^{-1}). The resulting charge is shown in Fig. S8.1 (a) blue data points. The charging state describing how much charge is left after the delayed discharging is shown in Fig. S8.1 (b). We observe an initial fast decay of the charging state which subsequently levels out. After 500 s and 1000 s, a respective 63 % and 55 % of the initial charge was left on the device. We also analyze delayed discharge when charged not with light but with an electric charging current (10.5 mA g^{-1}) in the dark (Fig. S8.1 (a) and (b) red data points). Interestingly, while under the chosen conditions a larger initial charge was present on the device (3.12 C g^{-1} for electric charging vs. 2.41 C g^{-1} for light charging), a delayed discharge decreases the charge output less than for light charging (after 500 s and 1000 s, a respective 81 % and 72 % of the initial charge was left on the device).

We rationalize the self-discharge with issues arising from sample preparation, such as pinholes, or more generally, insufficiently charge selective HTM performance to separate charges from the cathode and anode efficiently. Electrons and holes can thus slowly recombine via the internal layer, and discharge the system from the inside, i.e. by the electrode material adjacent to the HTM. The faster self discharge from light charging can be explained with light charging occurring more in the bulk of K-PHI and electric charging occurring more close to the junction to the substrate. Since the former charges K-PHI closer to the junction of the HTM and due to the low conductivity of K-PHI, light charging produces a slightly faster self discharge. In the electrically charged case, however, the material adjacent to the contact is charged first, thus hindering charge recombination through the material in the device volume.

9. Solar cell performance comparison 1 sun & 365 nm LED

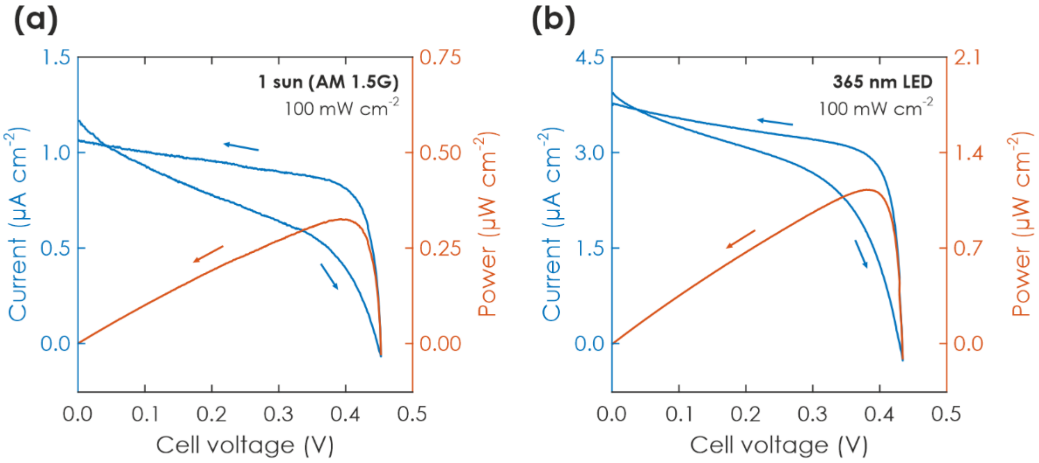

Fig. S9.1 | Comparison of IV curves with different light sources. (a) Current-voltage (blue) and power (red) curves (10 mV s^{-1}) of a solar battery sample in solar cell mode, illuminated with 1 sun. Reproduced from main text Fig. 2 (a). **(b)** Current-voltage (blue) and power (red) curves (10 mV s^{-1}) of a solar battery sample in solar cell mode, illuminated with a 365 nm LED (100 mW cm^{-2}; bandwidth of ca. 10 nm).

We discuss operation of the solar battery device in solar cell mode extensively in main text Section 2.2.1. Two different illumination sources are utilized for measurements presented in this work: Either artificial sunlight (1 sun) with AM 1.5 global standard (see Methods section for details) to provide real-world illumination conditions for operation of the solar battery, or an 365 nm LED (ca. 360-375 nm) to provide light which is below the bandgap of K-PHI (450 nm). Both light sources were operated at the same power setting (100 mW cm^{-2}). IV and power curves are shown in Fig. S9.1 and performance parameters are summarized in Table S9.1. Notably, the shape of IV and power curves looks very alike and open-circuit potential, potential at which maximum power is provided as well as the fill factor are very similar. However, as expected short-circuit current is significantly higher when operated with the LED (277 %) and the device produces much more power (248 %).

Table S9.1 | Performance parameter of IV curves. Summary of performance parameters of current-voltage and power curves of the solar battery, when illuminated either with artificial sunlight or a 365 nm LED. The fill factor (FF) is calculated via the backward sweep (OCP to 0 V) from open circuit potential (U_{OCP}), short circuit current (I_{SC}), and potential (U_{Pmax}) as well as current (I_{Pmax}) at which the device produces maximum power (P_{max}). IV curves are shown in Fig. S9.1.

**Illumination mode**	**U_{OCP}** (V)	**U_{Pmax}** (V)	**I_{sc}** (µA cm^{-2})	**I_{Pmax}** (µA cm^{-2})	**P_{max}** (µW cm^{-2})	**FF**
1 sun (100 mW cm^{-2})	0.45	0.39	1.07	0.828	0.326	0.68
365 nm LED (100 mW cm^{-2})	0.43	0.38	3.78	2.98	1.13	0.69

10. Comparison of solar battery performance with literature

Table S10.1 | Comparison of performance of several different solar battery materials. $\eta_{conversion}$ gives the solar-to-output efficiency defined as light energy input divided by energy from electric discharging, and $\eta_{round\ trip}$ gives the additional round-trip efficiency upon illumination, defined as electric energy from electric charging (under illumination) divided by electric energy from discharging.

Photo electrode	Counter electrode	Electrolyte	Battery type	$\eta_{conversion}$ (%)	Additional $\eta_{roundtrip}$ (%)	Cycling stability	Year Ref
**Photoanode design**							
K-PHI anode	PEDOT:PSS cathode	F8BT solid	battery	0.012*	94.1	50	*This work*
F8BT anode	ITO	Na_2SO_4/PVA gel	super capacitor	0.0017	-	-	2021 [11]
Na_xMoO_3 anode	MnO_2 cathode	Na_2SO_4 aqueous	half flow battery	-	-	8	2017 [12]
TiO_2@WO_3 anode[#]	I^-/I_3^- via Pt cathode	I^-/I_3^- in propyl-ene carbonate	half flow battery[#]	-	-	-	2019 [13]
TiO_2@carbon nitride anode	I^-/I_3^- cathode	I^-/I_3^- in ace-tonitrile	half flow battery[#]	-	-	-	2021 [14]
**Photocathode design**							
2D Perovskite† cathode	Li metal anode	$LiPF_6$	Li-ion battery	0.034	-	10	2018 [15]
TiO_2/Fe_2O_3 cathode	Li metal anode	$LiCF_3SO_3$ in TEGDME	Li-O_2 battery	-	86	100	2020 [16]
V_2O_3/P3HT/rGO cathode	Li metal anode	LiTFSI	Li-ion battery	0.22	57	35	2021 [17]
V_2O_3/P3HT/rGO cathode	Zn metal anode	$Zn(CF_3SO_3)_2$ aqueous	Zn-metal battery	1.2	-	25	2020 [18]

*: for illumination with AM1.5G, when only taking light below the bandgap into account (< 450 nm). For full AM1.5 G spectrum: 0.002 %. See SI Section 5 for details.

#: bifunctional composite electrode. Half flow battery means that the cathode is not a solid electrode but rather an electrolyte.

†: $(C_6H_9C_2H_4NH_3)_2PbI_4$ perovskite, abbreviated as CHPI.

11. References

1. S. Fleischmann, J. B. Mitchell, R. Wang, C. Zhan, D.-e. Jiang, V. Presser and V. Augustyn, *Chem. Rev.*, 2020, **120**, 6738-6782.
2. V. Augustyn, P. Simon and B. Dunn, *Energy Environ. Sci.*, 2014, **7**, 1597-1614.
3. F. Podjaski, J. Kroger and B. V. Lotsch, *Adv. Mater.*, 2018, **30**, 1705477.
4. Y. Shao, M. F. El-Kady, J. Sun, Y. Li, Q. Zhang, M. Zhu, H. Wang, B. Dunn and R. B. Kaner, *Chem. Rev.*, 2018, **118**, 9233-9280.
5. C. Costentin, T. R. Porter and J. M. Saveant, *ACS Appl. Mater. Interfaces*, 2017, **9**, 8649-8658.
6. C. Costentin and J. M. Savéant, *Chem. Sci.*, 2019, **10**, 5656-5666.
7. K. Sun, S. P. Zhang, P. C. Li, Y. J. Xia, X. Zhang, D. H. Du, F. H. Isikgor and J. Y. Ouyang, *J. Mater. Sci.: Mater. Electron.*, 2015, **26**, 4438-4462.
8. A. M. Bryan, L. M. Santino, Y. Lu, S. Acharya and J. M. D'Arcy, *Chem. Mater.*, 2016, **28**, 5989-5998.
9. A. V. Volkov, K. Wijeratne, E. Mitraka, U. Ail, D. Zhao, K. Tybrandt, J. W. Andreasen, M. Berggren, X. Crispin and I. V. Zozoulenko, *Adv. Funct. Mater.*, 2017, **27**, 1700329.
10. Sellam and S. A. Hashmi, *ACS Appl. Mater. Interfaces*, 2013, **5**, 3875-3883.
11. W. J. Dong, W. S. Cho and J. L. Lee, *ACS Appl. Mater. Interfaces*, 2021, **13**, 22676-22683.
12. S. N. Lou, N. Sharma, D. Goonetilleke, W. H. Saputera, T. M. Leoni, P. Brockbank, S. Lim, D. W. Wang, J. Scott, R. Amal and Y. H. Ng, *Adv. Energy Mater.*, 2017, **7**, 1700545.
13. Z. Wang, H. C. Chiu, A. Paolella, K. Zaghib and G. P. Demopoulos, *ChemSusChem*, 2019, **12**, 2220-2230.
14. L. He, X. Tang, Y. Li, L. Zhang and G. Cao, *Adv. Energy and Sustainability Res.*, 2021, **2**, 2100111.
15. S. Ahmad, C. George, D. J. Beesley, J. J. Baumberg and M. De Volder, *Nano Let.*, 2018, **18**, 1856-1862.
16. M. Li, X. Wang, F. Li, L. Zheng, J. Xu and J. Yu, *Adv. Mater.*, 2020, **32**, 1907098.
17. B. D. Boruah, B. Wen and M. De Volder, *Nano Letters*, 2021, **21**, 3527-3532.
18. B. D. Boruah, A. Mathieson, B. Wen, S. Feldmann, W. M. Dose and M. De Volder, *Energy Environ. Sci.*, 2020, **13**, 2414-2421.
